# Virtual Interviews: A Cross-Sectional Quality Improvement Project Aimed to Improve the Interview Process in an Internal Medicine Residency Program

**DOI:** 10.7759/cureus.34927

**Published:** 2023-02-13

**Authors:** Ram Prakash Thirugnanasambandam, Violeta Capric, Krunal H Patel, Harjinder Gill, Patrick Geraghty

**Affiliations:** 1 Internal Medicine, State University of New York Downstate Health Sciences University, Brooklyn, USA

**Keywords:** categorical cadidates, preliminary candidates, resident interaction, program website, virtual interview

## Abstract

Background: The residency interview is a crucial step that helps the program identify potential new trainees while the trainees find out more about the program. With the onset of the COVID-19 pandemic, it became essential to hold interviews virtually.

Objective: Here, we conducted a questionnaire-based study to identify areas of improvement in the virtual interview process in our program.

Methods: The study was conducted among the residency interview applicants of the 2022 match cycle. A questionnaire was sent via email to all applicants who were invited to our residency program for an interview. Out of the 600 applicants who were interviewed in our program, 230 applicants answered the survey, an 11-point questionnaire pertaining to the various aspects of the interview process.

Results: A virtual interview with an option of in-person is the most favored answer among the different groups, i.e., American Medical Graduates (AMGs), International Medical Graduates (IMGs) without need for a visa, and IMGs with a need for a visa, respectively, were, 37.5%, 42.8%, and 38.65%, respectively. An updated website with all required information was a top resource used by 95%, 84.5%, and 89.6% of the different groups of candidates. American medical graduates (32.50%) place high importance on resident interaction, while international medical graduates want the programs to focus on their website and provide more information about all aspects of the program during the virtual interview.

Conclusions: In the post-COVID-19 era, Internal Medicine programs will need to improve several aspects of virtual interviews while assessing what is important to candidates.

## Introduction

The residency interview is a crucial step that acts as the meeting point for the applicant and the residency program. It helps the program identify potential new trainees while the trainees find out more about the program and if they would like to train at the program for their residency. Coronavirus disease (COVID-19), while having a great impact on medicine, has also ushered in a new era of virtual interviews. The interviews season in 2020 was the first time programs across the country had to resort to the use of virtual interviews based on recommendations of the Coalition for physician accountability [1]. However, the use of virtual interviews is not entirely new and was previously used by specialties such as Urology, Obstetrics, Gynecology, and Orthopedics in their recruitment process. A literature search shows that the initial response from many applicants did not have a satisfactory interview experience [2,3]. However, with the widespread nature of the pandemic, it has now become essential to hold interviews virtually, keeping in mind the safety of everyone involved in the process as a top priority. While virtual interviews helped with overcoming a few hurdles during the pandemic (e.g., the impact of travel bans across the world), they also came with their downside, with the biggest issue being the inability of applicants to see the program in person [4].

International Medical Graduates (IMGs) are an important part of the continued Graduate medical education (GME) and hospitals across the country contributing to relieving the linguistic and cultural barriers to receiving healthcare [5]. The hurdles they meet begin long before application season, and they constantly work to overcome hurdles such as adapting to a different work environment, improving their communication skills, overcoming emotional distress, and struggling to afford the financial cost of applications and the interview season. The differentiation between the two groups would thus give appropriate information to program directors on possible changes that could be made in their interviews, keeping the needs of both the AMGs and the IMGs in mind. Here, we conducted a questionnaire-based study to help find out the potential areas of improvement in the virtual interview process in our program.

## Materials and methods

A questionnaire, which was designed to focus on key aspects of interviews, was sent via email to all applicants who were invited to the SUNY Downstate residency program for an interview. The questionnaire, which was designed to be anonymous, was sent after the national residency matching program (nrmp) rank list deadline submission day to prevent any bias in answering questions related to their interviews. The study was also Institutional Review Board (IRB) exempt as deemed by the IRB at SUNY Downstate. The number of candidates who were interviewed for the 2022 match at our program was approximately 600. Two hundred thirty candidates answered the questionnaire (Appendix) between March to July 2022. The 11-point questionnaire pertained to the various aspects of the interview process. Among the 230 respondents to our questionnaire, there were no candidates who had applied to primary care, and hence the subgroup has not been included in our analysis and discussion. Analysis was carried out using StataCorp. 2017. Stata Statistical Software: Release 15. College Station, TX: StataCorp LLC. Likert scales were converted to numerical scores (1-5), and Microsoft Excel (Redmond, USA) was utilized to generate graphs. Frequencies and percentages were used to summarize categorical variables. 

## Results

**Figure 1:**
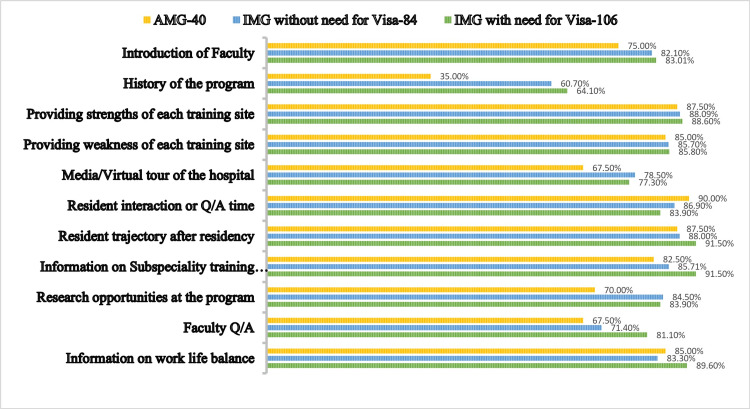
Comparison of the three different sub groups of candidates on their rating of aspects of the interview. Percentage is used for comparison values.

**Figure 2:**
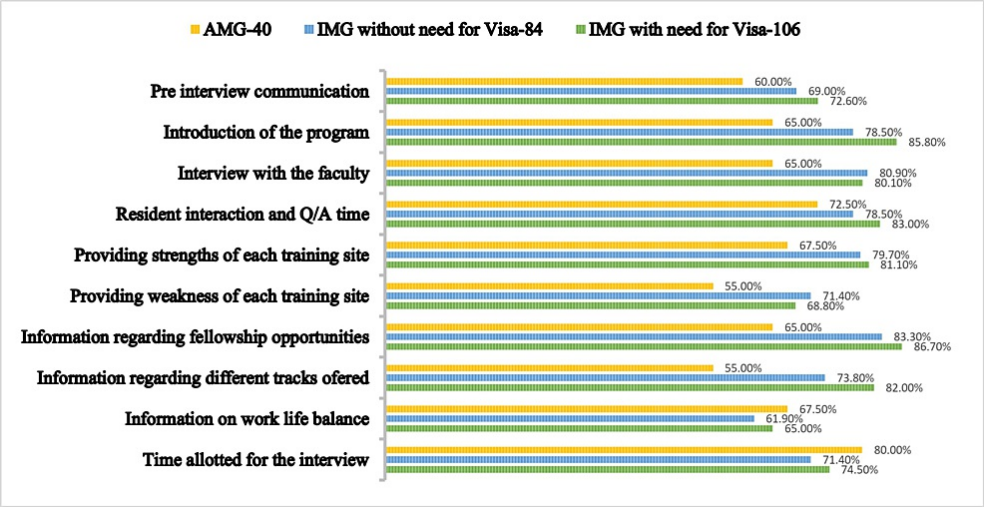
Comparison of the three different sub groups of candidates on their experience at SUNY Downstate on different aspects of the interview. Comparison values are given in percentage.

Among the 230 candidates, there were 40 AMGs, 84 IMGs without the need for a visa, and 106 IMGs with a need for a visa. More than 50% of candidates among all groups have indicated that they would like a virtual interview or a virtual interview with an option of in-person (Table 1). The majority of candidates among the three different subgroups (95%, 84.5%, and 89.6%, respectively) rated the program website as their top resource to utilize for their interviews, while the option of “speaking to current or past residents” is seen to be rated second among all groups (87.5%, 76.1%, and 87.7%, respectively) (Table 2). From a candidate’s perspective, while all aspects of a residency interview were rated highly, aspects such as resident trajectory, resident interaction, and introduction of faculty were deemed important by both AMGs and IMGs, with IMGs rating all of them higher (Figure 1). Our residency program scored well among IMGs in several aspects of the interview, such as information about fellowship opportunities and introduction to the program, with more than 85% of applicants rating their experience highly (Figure 2).

**Table 1:** Demographics of candidates, along with the preference for interviews Data are presented as number (N), and the percentage is given in brackets (percentage of the total number in the group. i.e., AMG, IMG without need for visa and IMG with a need for visa)

Total No. of respondents	AMG(N)	IMG without the need for a Visa(N)	IMG with the need for a Visa(N)	p-value
230	40 (17.3%)	84 (36.5%)	106 (46%)	
Preliminary	12 (30%)	11 (13%)	4 (3.7%)	<0.0001
Categorical	25 (62.5%)	67 (79.7%)	101 (95.2%)	<0.0001
Categorical and primary care	3 (7.5%)	6 (7.14%)	1 (0.94%)	<0.0643
No. of the respondents from different groups that said yes to having the option of virtual and in-person interviews	25 (62.5%)	57 (67.8%)	64 (60.3%)	<0.5624
Type of interview preferred				
In-person	12 (30%)	28 (33.3%)	28 (26.4%)	<0.5823
Virtual	13 (32.5%)	20 (23.8%)	37 (34.9%)	<0.2438
Virtual with an option of in-person	15 (37.5%)	36 (42.8%)	41 (38.6%)	<0.7918

**Table 2:** Resources used for the interview Data are presented as number (N), and the percentage is given in brackets (Brackets: percentage of the total number in the group, i.e., AMG, IMG without need for visa and IMG with a need for visa).

Resources used for the Interview	AMG-40(N) (Rated it a 4 or 5)	IMG without the need for a Visa-84(N) (Rated it a 4 or 5)	IMG with the need for a Visa-106(N) (Rated it a 4 or 5)	p-value
Program website	38 (95%)	71 (84.5%)	95 (89.6%)	<0.2086
Virtual open house	26 (65%)	57 (67.8%)	76 (71.6%)	<0.7008
Other internet sources	21 (52.5%)	47 (55.9%)	68 (64.1%)	<0.3355
Speaking to past or current residents	35 (87.5%)	64 (76.1%)	93 (87.7%)	<0.0782

**Figure 3:**
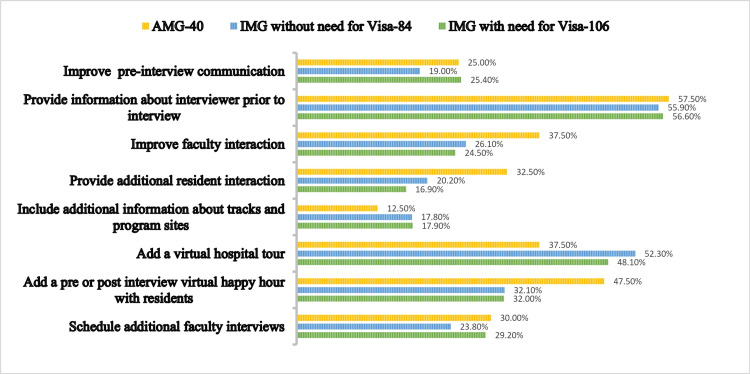
Comparison of the three different sub groups of candidates on steps to improve the interview experience at SUNY Downstate. Comparison values are given in percentage.

**Figure 4:**
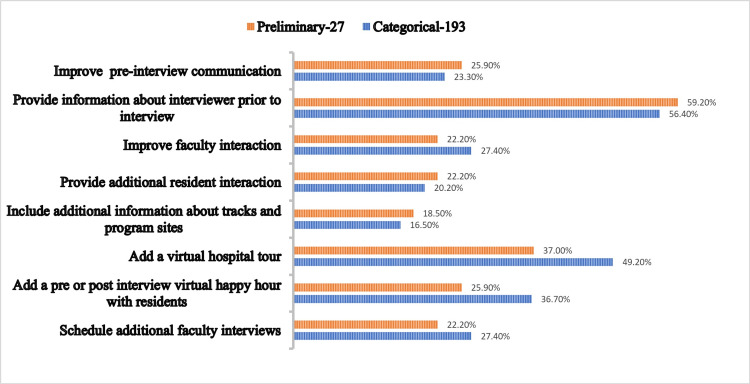
Comparison between preliminary and categorical candidates on steps to improve the interview experience at SUNY Downstate. Comparison value are given in percentage

When given options to help us improve the interview process AMGs chose options such as improving faculty interaction, provident additional resident interaction, and providing a pre- or post-interview virtual happy hour, whereas IMGs chose to include additional information about different tracks offered and add a virtual tour of the hospital above AMGs (Figure 3). Preliminary candidates wanted the program to improve the pre-interview communication and provide information about the interviewers before the interview, whereas categorical applicants wanted the program to focus on improving faculty interaction during interviews, adding a virtual hospital tour and a virtual happy hour, and scheduling additional interviews (Figure 4).

## Discussion

The study aimed to improve the interview process at our program and specifically addressed the aspects of the virtual interview, which has now become. An 11-point questionnaire was sent to the 600 applicants who interviewed at our program for the 2022 match cycle. Two hundred and thirty candidates responded to the survey. Table 1 shows the demographics of the candidates. We divided the candidates into three different categories: 1: American medical graduates (AMGs), 2: International medical graduates (IMGs) without a need for visa, and 3: International medical graduates with a need for visa sponsorship. The differentiation of candidates was performed to show the involvement of all types of candidates and to also ascertain if different categories of candidates had certain areas of preference for improvement in the interview process.

Table 1 shows the demographics along with preferences in the type of interview among different groups. As seen in multiple studies and papers, the state of New York is among the states with a high intake of IMGs, and it is no coincidence that it is reflected in our study [6-8]. We divided the candidates based on the program they applied to. This differentiation was done to analyze if there were different needs that we need to focus on among candidates applying to the different tracks of programs available to our residency program. The different tracks offered in our program include Preliminary, Categorical, and Primary care. The survey was sent to all candidates who interviewed with our categorical program alone. However, 10 candidates have indicated that they applied to both the categorical and primary care tracks. In a study conducted by Chadaga AR et al., it has been shown that preliminary candidates have different preferences as compared to the categorical candidates in terms of the overall duration of interview days, interview time, and interactions with the house staff [9]. This could be reflective of the fact that they would prefer to spend more time on the interviews for their chosen specialty. Hence, we decided to take note of their preferences in improving the interview process in question eight.

In response to the type of interview preferred (Table 2), we see that a virtual interview with an option of in-person is the most favored answer among the different groups (AMGs and IMGs) with 37.5%, 42.8%, and 38.65%, respectively. This is perhaps reflective of the mindset of applicants keeping in mind the effects of the pandemic as well as having seen the benefits of the newly introduced virtual interview process. Different studies show that program directors (PDs) and applicants preferred the virtual interview process over the traditional in-person interview [10-12]. This trend toward favoring virtual interviews is possibly due to the high financial costs involved by both parties in the interview process. The cost of recruitment for internal medicine programs averages $148,000 per season per program [13]. In the study by Robinson KA et al. [10], the median cost of $600 per in-person interview seems to place a high degree of financial burden on the applicant, and it is only logical that the majority would choose against in-person interview. In the same study, both PDs and applicants felt that in-person interviews strengthen the connection between the candidate and the program. Applicants also voted for an in-person option in their study as opposed to PDs who did not. Overall, we can conclude that virtual interviews could have a higher benefit, and the option of having in-person can be a choice to be discussed by each program.

Utilizing the program website topped the choices, followed by speaking to current or past residents among the AMGs and IMGs when asked about resources used by them to prepare for the interview process. The program website is the obvious first step in understanding the program among all groups of applicants and sets a tone of interest among applicants in choosing to apply to a program. The most important information sought after would be details of the program itself, faculty and resident profiles, and research opportunities [14,15]. IMGs understand the recruitment process of a program by looking through the resident profiles, and it is an important factor to be noted by PDs when updating their websites. Chadaga AR et al. also brought into focus the importance of applicants directly speaking to residents and being interviewed by chief residents. Their study showed that AMGs preferred speaking to residents over IMGs though it is seen that IMGs preferred a time frame of fewer than 60 minutes to interact with residents as opposed to having no interaction at all. However, in our survey, both groups had similar interests, and this is a potential area for PDs to focus more on during their virtual interview process.

Programs routinely use aspects such as application features/programs, personal statements, recommendation letters, curriculum vitae, and interviews to assess the communication and interpersonal skills, knowledge base, and experience of the applicant [16]. We wanted to know how the applicants did the same for the program they interview at. The survey asked the applicants to rate the various aspects of the interview on a scale of 1 to 5, with a rating of 4 or 5 considered very important to them. Figure 1 shows the importance placed by AMGs and IMGs on 11 aspects of the interview, which were chosen by us after a discussion on the interview process. The study by Phitayakorn R et al. showed that applicants regard the ability of the program to prepare residents for future training or position, the morale of residents, faculty at the program, and patient diversity to be the most important aspects to candidates [17]. This is reflected in our survey, where aspects such as resident trajectory, resident interaction, and introduction of faculty were deemed important by both AMGs and IMGs, with IMGs rating all of them higher. A similar study by Gaeta TJ et al. showed that applicants considered the presentation of the curriculum, information about the hospital and its affiliates, faculty and resident information, and research activities to be important as well [18]. Surprisingly, the virtual tour of the hospital ranked low even though it was thought applicants would like to see the features of the various teaching sites involved.

Our program scored well among IMGs in several aspects of the interview (Figure 2), such as information about fellowship opportunities and introduction to the program, with more than 85% of applicants rating their experience highly. The aspects such as resident interaction or Q/A time, interviews with faculty, information on the different tracks offered at the program and strengths of each training site chosen by more than 80% of IMGs. AMGs rated the time allotted for the interview higher than other aspects, followed by resident interaction or Q/A time. However, the number of respondents in the AMG group was smaller, so it might be difficult to compare groups.

We compared different groups on the options they chose to help us improve the interview process (Figure 3). AMGs chose options such as improving faculty interaction, provident additional resident interaction and providing a pre- or post-interview virtual happy hour over IMGs. Whereas IMGs chose to include additional information about different tracks offered and add a virtual tour of the hospital above AMGs. This may reflect the importance of resident interaction placed by AMGs and more program information required by the IMGs, which are to be noted by the PDs. Another important comparison was done by looking at the preliminary vs. categorical groups (Figure 4). Preliminary applicants wanted the program to improve the pre-interview communication and provide information about the interviewers prior to the interview. Categorical applicants wanted the program to focus on improving faculty interaction during interviews, adding a virtual hospital tour, the addition of a virtual happy hour and scheduling additional interviews. Both groups have shown interest in different aspects, which was expected by our team. 

Finally, 171 (74.34%) of candidates responded with a 4 or 5 on a scale when asked if they would recommend our program to a junior or a colleague at the next match cycle. Two hundred and seventeen (94.35%) of candidates did not experience any technical difficulties during their interview. 

The limitations of the study include the following: i) the survey was sent to all applicants who interviewed at the program, so there is a potential for bias in their answers; however, we sent the survey after the rank order list deadline to avoid any kind of bias ii) the demographics of the candidates who participated in the survey is not available, but the survey avoided questions about demographics as we did not think it was an important criteria to group candidates answers based on their gender or race.

## Conclusions

Virtual interviews are likely to become the norm for all residency programs across the country despite the COVID-19 pandemic being less pathogenic. Applicants and PDs view the benefits of virtual interviews to outweigh the benefits of the in-person interview. Programs must work together with the applicants to help each other find their most suitable match. Programs must update their website with all required information as required by the applicants if they want to attract the most competitive applications. Applicants have consistently placed aspects such as presentation of the curriculum, information about the hospital and its affiliates, faculty and resident information, and research activities as important aspects in their evaluation of a program, and hence PDs must work to provide information on all these categories. Our institution undertook this survey to address aspects ranked high by the respondents to our survey. AMGs and our institution place high importance on resident interaction, which can be made possible by having more resident interaction time with virtual happy hours or allotting more time during the interview for the applicants to talk to current residents. PDs can help IMGs by updating the program website and providing more information about all aspects of the program during the virtual interview. Finally, programs can be applicant-centric by focusing on aspects considered important by preliminary applicants in addition to their categorical counterparts.

## Figures and Tables

**Figure 5:**
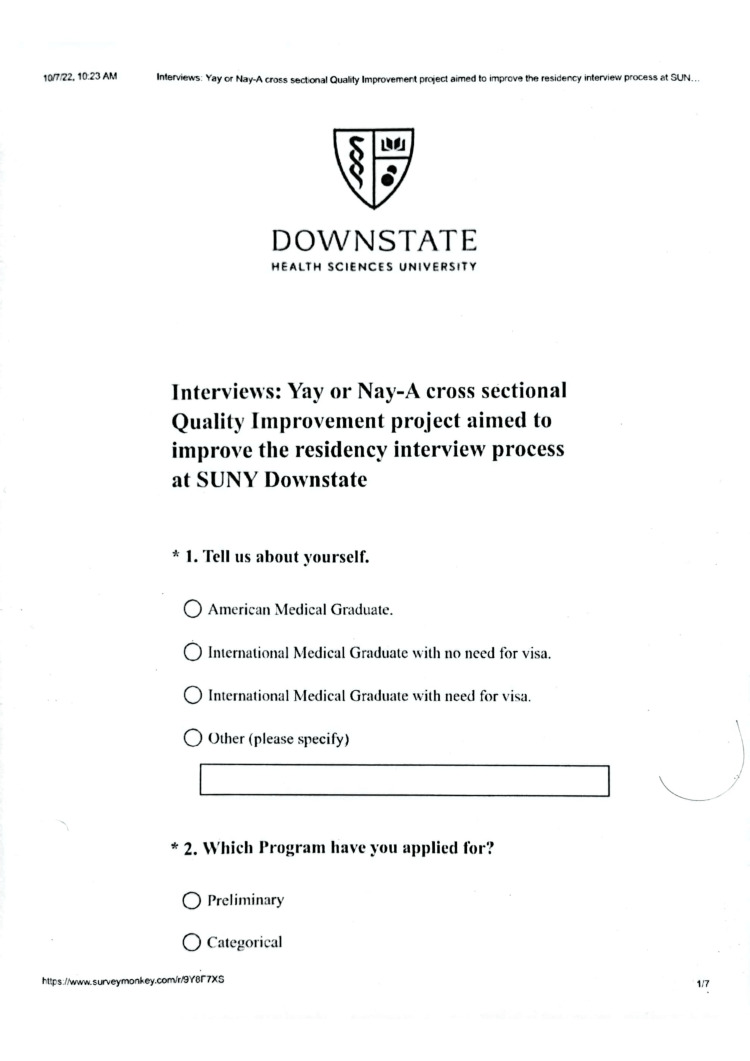
Questionnaire Questions 1 and 2

**Figure 6:**
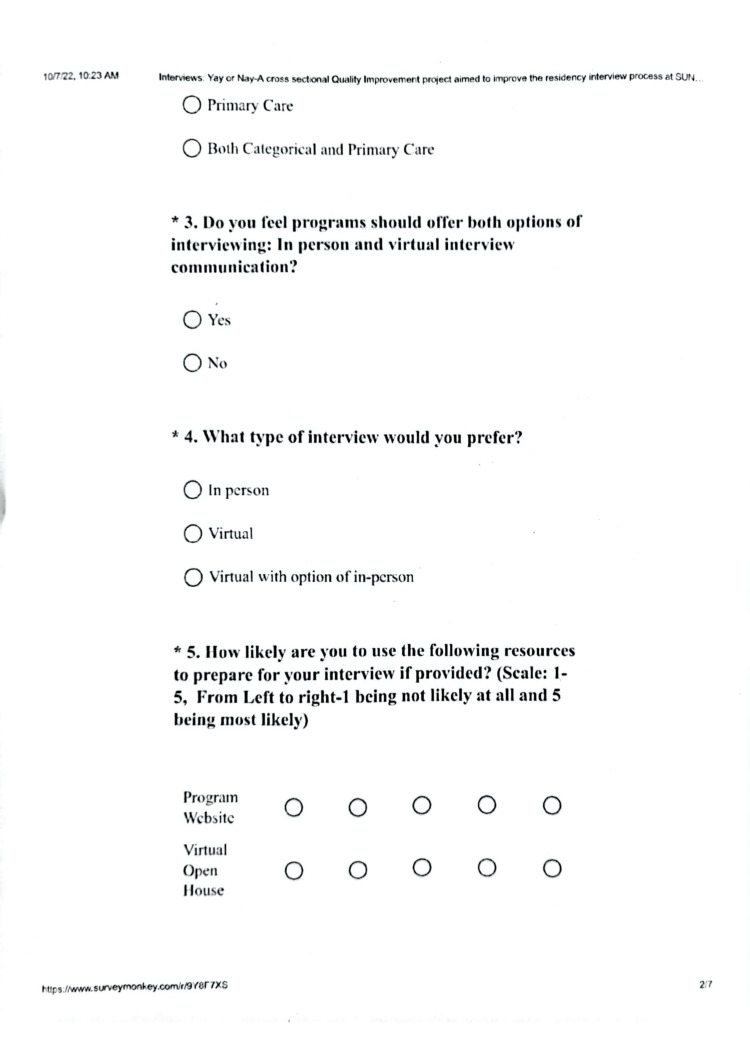
Questionnaire Questions 3, 4, and 5.

**Figure 7:**
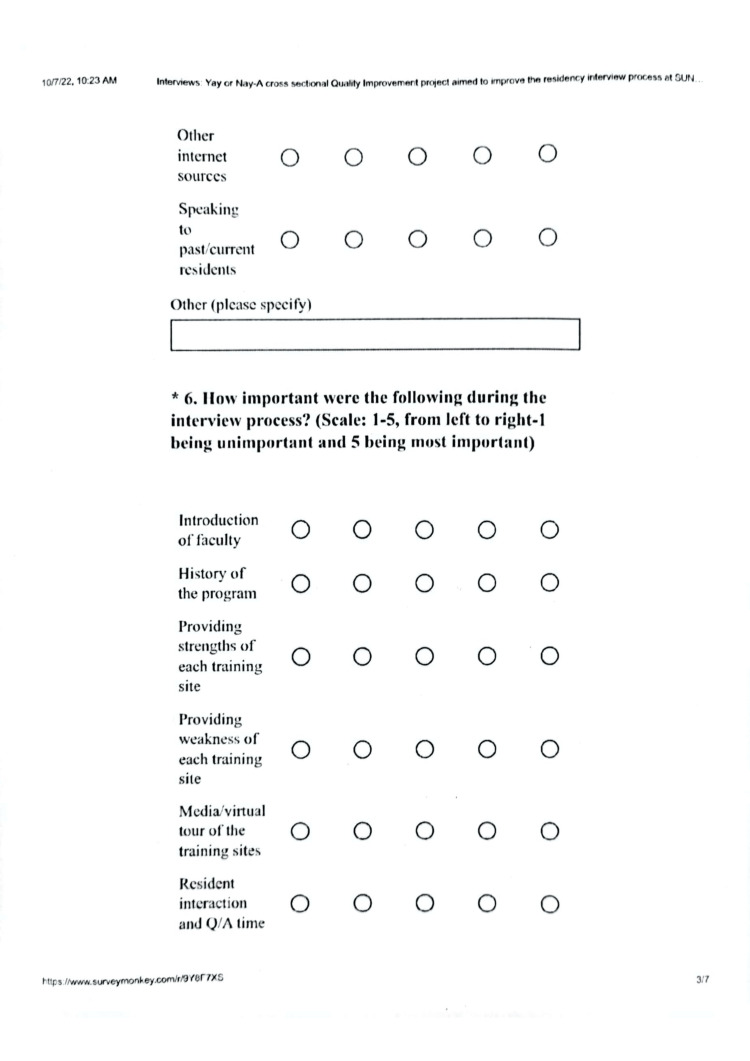
Questionnaire Question 6.

**Figure 8:**
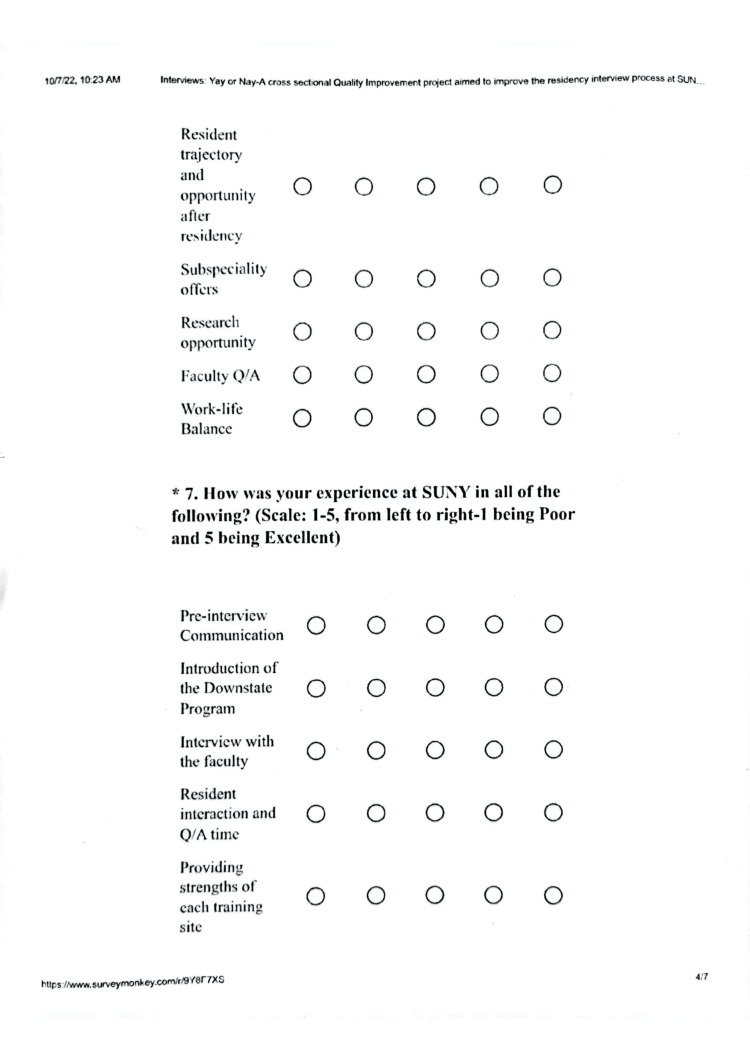
Questionnaire Question 7.

**Figure 9:**
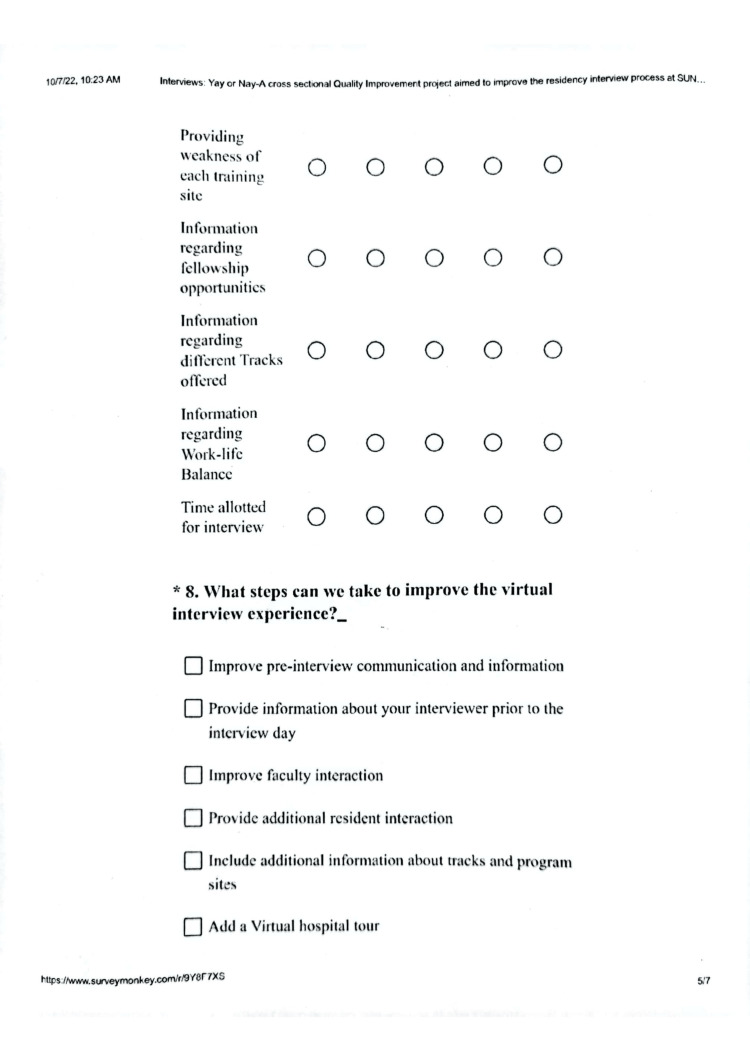
Questionnaire Question 8.

**Figure 10:**
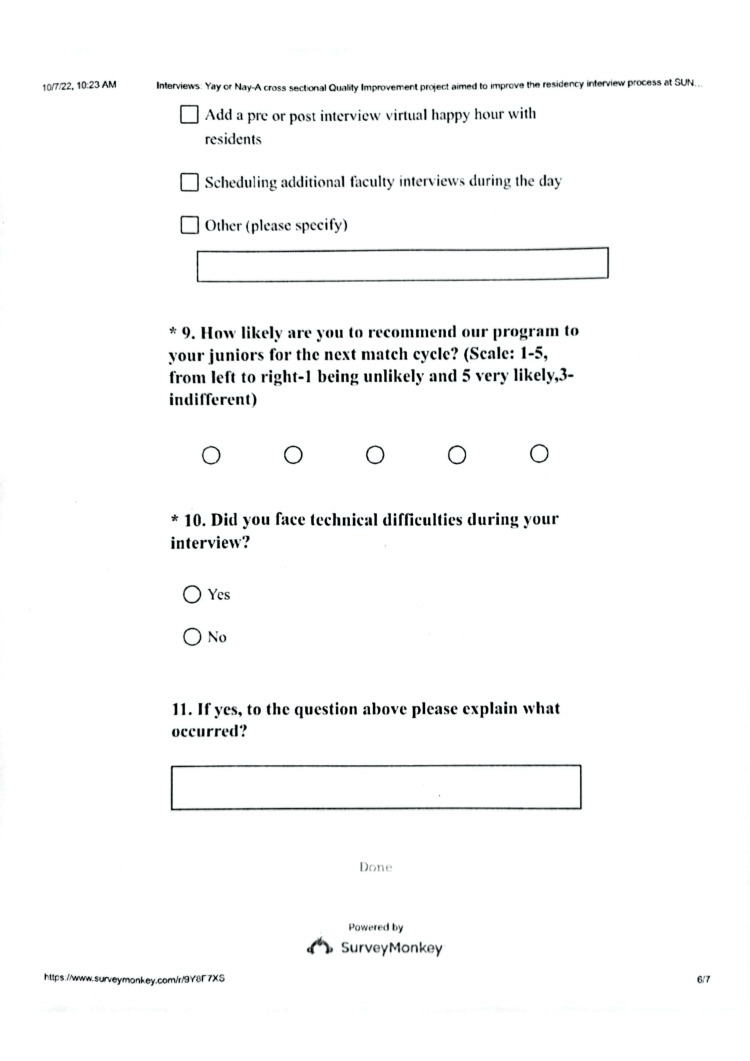
Questionnaire Questions 9, 10, and 11.

## Appendices

The questions from the questionnaire are listed in Figures 5-10.

## References

[1] Bhardwaj P, Kleiber GM, Baker SB, Fan KL (2020). Applying to Residency in the COVID-19 Era: Virtual Interview Tips for Success. Plast Reconstr Surg Glob Open.

[2] Breitkopf DM, Green IC, Hopkins MR, Torbenson VE, Camp CL, Turner NS 3rd (2019). Use of Asynchronous Video Interviews for Selecting Obstetrics and Gynecology Residents. Obstet Gynecol.

[3] Shah SK, Arora S, Skipper B, Kalishman S, Timm TC, Smith AY (2012). Randomized evaluation of a web based interview process for urology resident selection. J Urol.

[4] Burrell D The Virtual Residency Interview Season: Are You Ready?. Here We Go Again! R I Med J.

[5] Murillo Zepeda C, Alcalá Aguirre FO, Luna Landa EM, Reyes Güereque EN, Rodríguez García GP, Diaz Montoya LS (2022). Challenges for International Medical Graduates in the US Graduate Medical Education and Health Care System Environment: A Narrative Review. Cureus.

[6] Scheitler KM, Lu VM, Carlstrom LP, Graffeo CS, Perry A, Daniels DJ, Meyer FB (2020). Geographic Distribution of International Medical Graduate Residents in U.S. Neurosurgery Training Programs. World Neurosurg.

[7] Ramos-Rodriguez AJ, Timerman D, Kyriacou MI, Martin RF (2019). A strategic evidence-based framework for international medical graduates (IMGs) applying to dermatology residence in the United States: a literature review. Dermatol Online J.

[8] Salsberg ES, Wing P, Dionne MG, Jemiolo DJ (1996). Graduate medical education and physician supply in New York State. JAMA.

[9] Chadaga AR, Villines D, Krikorian A (2018). Medical student preferences for the internal medicine residency interview day: A cross-sectional study. PLoS One.

[10] Robinson KA, Shin B, Gangadharan SP (2021). A Comparison Between In-Person and Virtual Fellowship Interviews During the COVID-19 Pandemic. J Surg Educ.

[11] Hemal K, Sarac BA, Boyd CJ, Runyan CM, Gosman AA, Janis JE (2021). Applicant Preferences for Virtual Interviews: Insights from the 2020-21 Integrated Plastic Surgery Application Cycle. Plast Reconstr Surg Glob Open.

[12] Aljamaan F, Alkhattabi F, Al-Eyadhy A (2021). Faculty Members’ Perspective on Virtual Interviews for Medical Residency Matching during the COVID-19 Crisis: A National Survey. Healthcare (Basel).

[13] Wolff M, Burrows H (2021). Planning for Virtual Interviews: Residency Recruitment During a Pandemic. Acad Pediatr.

[14] Weston JA, David EA (2021). Program Directors Update Your Website Because It Matters…Now More Than Ever. Ann Thorac Surg.

[15] Rajaram R, Abreu JA, Mehran R, Nguyen TC, Antonoff MB, Vaporciyan A (2021). Using Quality Improvement Principles to Redesign a Cardiothoracic Surgery Fellowship Program Website. Ann Thorac Surg.

[16] Butts AR, Smith KM (2015). Application and interview features used to assess applicant qualifications for residency training. Hosp Pharm.

[17] Phitayakorn R, Macklin EA, Goldsmith J, Weinstein DF (2015). Applicants' Self-Reported Priorities in Selecting a Residency Program. J Grad Med Educ.

[18] Gaeta TJ, Birkhahn RH, Lamont D, Banga N, Bove JJ (2005). Aspects of residency programs' web sites important to student applicants. Acad Emerg Med.

